# Comparison of Ultrasound-Microwave-Assisted and Hot Reflux Extractions of Polysaccharides from *Alpinia officinarum* Hance: Optimization, Characterization, and Antioxidant Activity

**DOI:** 10.3390/molecules30143031

**Published:** 2025-07-19

**Authors:** Haibao Tang, Baogang Zhou, Mengge Sun, Yihan Wang, Ran Cheng, Tao Tan, Dongsheng Yang

**Affiliations:** 1 School of Life Science, Zhuhai College of Science and Technology, Zhuhai 519090, China; tanghb23@mails.jlu.edu.cn (H.T.); 18043217471@163.com (B.Z.); sunmg22@mails.jlu.edu.cn (M.S.); w18904411011@outlook.com (Y.W.); chengran24@mails.jlu.edu.cn (R.C.); 2 School of Life Science, Jilin University, Changchun 130012, China

**Keywords:** *Alpinia officinarum* hance polysaccharide, extract optimization, structural characterization, structure–activity relationship, antioxidant activity

## Abstract

*Alpinia officinarum* Hance exhibits various bioactivities, with polysaccharides being one of its key bioactive components. However, the relationship between the structural characteristics of these polysaccharides and their bioactivities remains unclear and underexplored. In this study, to optimize the extraction process, a Response Surface Methodology-based design combined with single-factor experiments was applied to determine the optimal conditions for the ultrasonic-microwave-assisted extraction of polysaccharides from *A. officinarum*. The primary structural characteristics and antioxidant activities of two polysaccharide fractions, PAOR-1 extracted by ultrasonic-microwave-assisted extraction and PAOR-2 extracted by hot reflux extraction (HRE), were systematically compared. The optimal extraction conditions, including a liquid–solid ratio of 1:50, extraction time of 19 mins, and ultrasonic power of 410 W, yielded a maximum polysaccharide extraction rate of 18.28% ± 2.23%. The extracted polysaccharides were characterized as acidic polysaccharides with a three-dimensional structure. PAOR-1 and PAOR-2 have different monosaccharide compositions, surface morphologies, and thermal stabilities. The antioxidant activity in vitro studies suggest that PAOR-1 may have higher antioxidant activity than PAOR-2 due to its higher content of uronic acids, lower relative molecular mass, and a more closely packed spatial configuration. These findings provide a theoretical basis for the development and utilization of AOR.

## 1. Introduction

*Alpinia officinarum* Hance (AOR), a member of the Zingiberaceae family, is predominantly found in Guangdong, Hainan, and Taiwan in China [1]. Traditionally used in Chinese medicine, AOR is considered warm in nature and is associated with the spleen and stomach meridians, and is often applied for treating gastrointestinal disorders [2]. AOR has demonstrated various bioactivities, including antioxidant, as well as butyrylcholinesterase inhibition, as validated by computational and in vitro studies [3]. AOR is widely used in clinical studies due to its compatibility with various medicinal formulations, and to date, 337 compounds have been found in its rhizomes containing flavonoids, diarylheptanoids, polysaccharides, phenylpropanoids, and essential oils [4]. The research highlights its antispasmodic [5], anti-inflammatory [6], antioxidant [7], antitumor [8], antibacterial [9], and hypoglycemic effects [10], suggesting significant therapeutic potential.

Polysaccharides, composed of various monosaccharides linked by glycosidic bonds [11], are essential natural macromolecular polymers with crucial roles in medicine [12], food [13], packaging [14], and targeted drug delivery [15]. As one of the key active ingredients in AOR, its polysaccharides contribute to immune enhancement and metabolic regulation and exhibit notable antioxidant, anti-hepatocarcinoma, and hypolipidemic activities, showing promise for medical applications. However, studies on the extraction of AOR polysaccharides remain limited, with most research focusing on traditional hot-water extraction [8]. While some preliminary structural studies have been conducted, a comprehensive investigation into the optimization of extraction methods, such as ultrasonic-microwave-assisted extraction (UMAE) and polysaccharide and antioxidant comparison, is still lacking. Therefore, this study aims to further explore the activity of this polysaccharide and its value.

Currently, HRE is the most popular but the most expensive extraction method for polysaccharides [16], though it has limitations, such as a low extraction efficiency, prolonged duration, and potentially structurally degrades polysaccharides [17]. To address these drawbacks, novel extraction techniques were researched, including ultrasound-assisted extraction (UAE) [18], microwave-assisted extraction (MAE) [19], ultra-high-pressure extraction (UPE) [20], supercritical fluid extraction (SFE) [21], ultrasound-assisted enzymatic extraction (UAEE) [22], and ultrasonic-microwave-assisted extraction (UMAE) [23]. Between these, UMAE combines the benefits of UAE—with a shorter extraction time and higher efficiency—along the advantages of MAE, where microwave irradiation enhances dipole rotation and increases thermal energy, resulting in the production of polysaccharides [24]. This study is the first to explore the use of UMAE on AOR polysaccharides, aiming to maximize their yield while preserving bioactivity.

Accumulating evidence has established a close association between free radicals and the human aging process [25]. In recent decades, antioxidants have garnered increasing research attention due to their potential roles in aging intervention and related diseases. Among them, natural plant-derived antioxidants have emerged as promising candidates, primarily attributed to their advantageous profiles, including low toxicity, high chemical stability, excellent biosafety, and relatively easy accessibility to bioactive components. [26]. Excessive free radicals attack biological macromolecules in cells and cause oxidative stress [27,28]. At present, natural antioxidants prepared from polysaccharides are gradually becoming well known in the market [29]. As a medicine and food homologous plant, AOR has a high polysaccharide content. At present, some researchers have found that AOR polysaccharides display certain antioxidant activities [8]. In this paper, the UMAE of polysaccharides from AOR was optimized by RSM, and the structures and biological activities of the polysaccharides obtained by UMAE and HRE methods were compared. The aim was to provide a theoretical basis for the efficient extraction and quality evaluation of AOR polysaccharides.

## 2. Materials and Methods

### 2.1. Materials and Reagents

The dried rhizomes of *A. officinarum* were purchased from a licensed herbal market in Guilin City, Guangxi Zhuang Autonomous Region, China (a major producing area of *A. officinarum*) in July 2024. D-glucose and concentrated sulfuric acid were supplied by Sinopharm Chemical Reagent Co., Ltd. (Shanghai, China). A total of 30% hydrogen peroxide (H_2_O_2_), ferrous sulfate heptahydrate, biphenyl triol, potassium persulfate, salicylic acid, potassium ferricyanide, trichloroacetic acid, potassium chloride, ferric chloride, and ethanol (supplied by Sinopharm Chemical Reagent Co., Ltd.) were used. Sodium acetate trihydrate, hydrochloric acid, citric acid, DPPH, ABTS, ascorbic acid, and DEAE-52 nitrocellulose column (supplied by Shanghai Yuanye Biotechnology Co., Ltd., Shanghai, China) were used in the experiment. All standards (e.g., D-glucose, concentrated sulfuric acid) were of analytical reagent (AR) grade. Abbreviations: min = minutes, h = hours, and sec = seconds.

### 2.2. Extraction and Purification of A. officinarum Polysaccharides

#### 2.2.1. Extraction, Purification, and One-Factor Experiment for Polysaccharides

**Figure 1:**
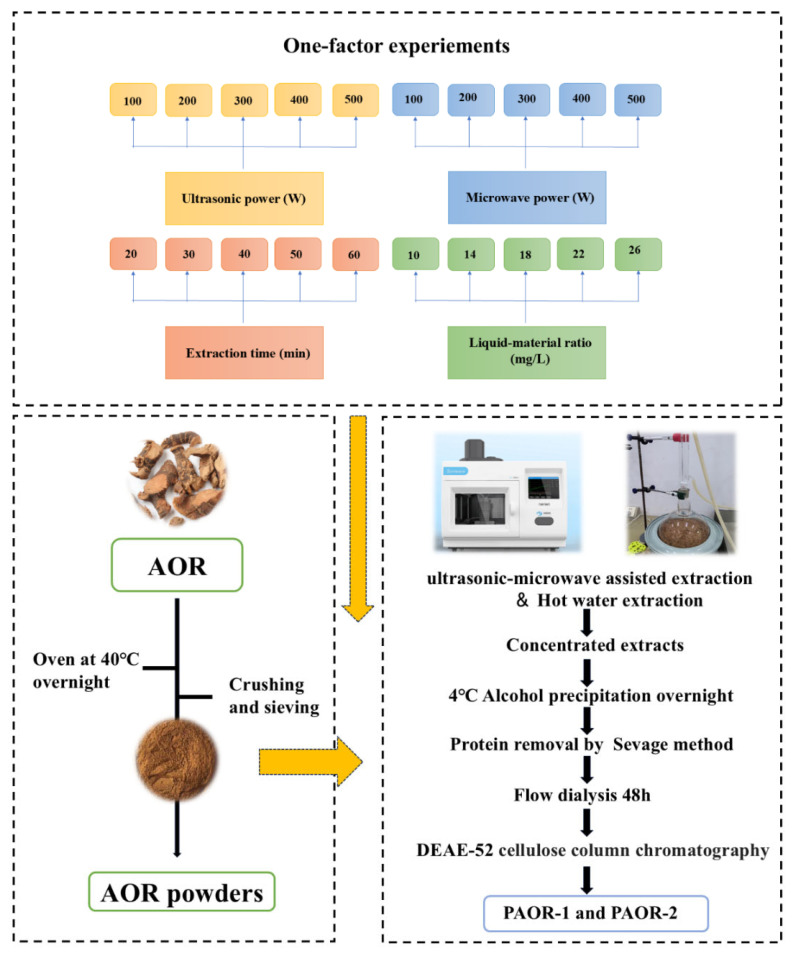
The procedure of the extraction method, and purification of PAOR-1 and PAOR-2.

Weigh 500 g of the *A. officinarum* rhizome, de-grease it in ethanol, and dry in an oven (DHG-9240A) until attaining a constant weight. The AOR rhizome was soaked in ethanol for 12 h at room temperature (approximately 25 °C). After de-greasing, the samples were dried in an oven at 60 °C until a constant weight was obtained. Regarding the AOR powder, for each extraction experiment using both the UMAE and HRE methods, precisely 10 g of AOR powder (with a particle size of 80 mesh, achieved by grinding with a high-speed universal pulverizer and sieving through standard test sieves) was weighed. For the UMAE process, a microwave–ultrasound combined extractor (model: XH−300A, manufacturer: Beijing Xianghu Technology Development Co., Ltd., Beijing, China) was employed. The instrument was set to an ultrasonic-power constant mode, where the ultrasonic power remained stable throughout the extraction. The ultrasonic parameters were set as 2 s on and 4 s off, and microwave and ultrasound were applied simultaneously during the extraction. When performing protein removal by the Sevage method, a mixture of chloroform and n-butanol was prepared at a volume ratio of 4:1. The Sevage reagent was added to the polysaccharide solution at a volume ratio of 1:5 (Sevage reagent:polysaccharide solution) [30]. Subsequently, the mixture was shaken vigorously for 20 min using a horizontal shaker (model THZ-3, Jiangsu Keda Instrument Co., Ltd., Suzhou, China) at 200 rpm, and then allowed to stand for 30 min to facilitate phase separation. The denatured protein layer at the interface was removed. This process was repeated 5 times until no protein layer appeared at the interface, indicating the completion of protein removal. Additionally, the protein removal operation using the Sevage method was carried out for 4 rounds in total. Finally, the purified polysaccharide obtained by vacuum freeze-drying after water dialysis for about 24 h was purified by a DEAE-52 nitrocellulose column to obtain PAOR-1 by the UMAE extraction method and PAOR-2 by the hot-water extraction method [30]. The one-factor experiment conditions for extraction and purification are depicted in Figure 1.

#### 2.2.2. Optimization of *A. officinarum* Polysaccharide Extraction

This experiment explored the interaction between three different factors and used the Response Surface Methodology (RSM) to screen out the best extraction process. A three-level experiment, including a solid–liquid ratio, extraction time, and ultrasonic power, was designed, based on the Box–Behnken Design (BBD). The experimental outcomes were employed to independently define the three levels (1, 0, −1) for each factor. The factors and levels of the RSM are presented in Table 1. For the optimization and statistical analysis of the predictive model, Design-Expert 13.0 software (Stat-Ease Inc., Minneapolis, MN, USA) was employed to generate the BBD matrix, perform regression analysis, and visualize response surface plots. Model fitting was evaluated using key statistical criteria, including the coefficient of determination (R^2^), adjusted coefficient of determination (Adj R^2^), and the F-value from the analysis of variance (ANOVA). A high R^2^ and Adj R^2^ (consistent with R^2^) indicated the good fit of the model to the experimental data.

Based on the principle of the BBD combination, the response surface optimization experiment shown in Table 2 was designed with A, B, and C response factors and the anthocyanin content as response values.

### 2.3. Carbohydrate and Total Proteins Determinations

Using the phenol–sulfuric acid method to measure carbohydrates [28]. The determination of the total protein content in two polysaccharide extracts was performed by using the Thomas Brilliant Blue kit [29]. 

### 2.4. Ultraviolet (UV) and Fourier-Transform Infrared Spectroscopy (IR) Spectra Analyses

The ultraviolet spectra of PAOR-1 and PAOR-2 were determined by a Shimadzu 1120 (Shimadzu Corporation, Kyoto, Japan) spectrophotometer in the wavelength range of 200–600 nm. The FT-IR spectra of PAOR-1 and PAOR-2 were determined by IRPrestige-21 (Shimadzu Corporation, Kyoto, Japan) in the range of 4000–400 cm^−1^ [31]. 

### 2.5. Determination of Monosaccharide Composition

Approximately 10 mg of polysaccharide was hydrolyzed with 1 mL of trifluoroacetic acid (TFA) at 108 °C for 4 h, neutralized with NaOH, and derivatized with 0.5 M 1-Phenyl-3-methyl-5-pyrazolone (in 0.3 M NaOH) at 65 °C for 1 h. After neutralization (0.3 M HCl) and chloroform extraction (×4), the supernatant was filtered (0.22 μm). The derivatives were separated using an Agilent 1260 HPLC system (Agilent Technologies, Santa Clara, CA, USA) with an Agilent Eclipse XDB-C18 column (4.6 mm × 250 mm, 5 μm). The mobile phase consisted of 83% 0.05 M potassium dihydrogen phosphate buffer (pH 6.8) and 17% acetonitrile, delivered at a flow rate of 1.0 mL/min. The detection wavelength was 254 nm, the column temperature was 28 °C, and the flow rate was 1 mL/min [32].

### 2.6. Molecular-Weight Determination

The polysaccharide sample solution was filtered through a 0.22 μm pore-size membrane prior to gel permeation chromatography (GPC) analysis. The GPC system consisted of an Agilent 1100 Series HPLC (Agilent Technologies, Santa Clara, CA, USA) equipped with a TSK-gel G3000 PWXL (Agilent Technologies, Santa Clara, CA, USA) column (7.8 mm × 300 mm). Isocratic elution was performed using a 0.05 M sodium sulfate solution as the mobile phase at a flow rate of 0.5 mL/min. The column temperature was maintained at 40 °C. The chromatographic column mainly used different dextran standards to create a standard curve, and then used log (Mw) as the abscissa and the retention time as the ordinate to calculate the relative molecular mass of the sample [32].

### 2.7. Microscopy Analyses

The polysaccharides were weighed and sprayed with gold in a vacuum state using an ion sputter coater (model: EMITECH K550X) to form a 10–20 nm thick conductive layer, then observed under a scanning electron microscope (Apreo2, Thermo Fisher Scientific, Waltham, MA, USA) at an accelerating voltage of 10 KV and magnifications of 200×, 2000×, and 10,000× to observe the surface morphology of the polysaccharide samples. For atomic force microscopy (AFM, AFM5500M, Hitachi High—Tech, Beijing, China) analysis, the polysaccharide samples were dissolved in ultrapure water to prepare a 0.1 mg/mL solution, and 5 μL of the solution was dropped onto a freshly cleaved mica sheet, dried at room temperature (25 ± 2 °C) for 24 h under dust-free conditions. The surface morphology of PAOR-1 and PAOR-2 was observed in the tapping mode with a silicon nitride probe at a scan rate of 1 Hz [8].

### 2.8. Thermal Analyses

An appropriate amount of polysaccharide samples was weighed, and their thermal properties were characterized by thermogravimetric analysis (TGA) using a thermogravimetric analyzer [33]. The heating rate was 10 °C/min, with the temperature range set from 30 to 560 °C under a nitrogen atmosphere to minimize oxidative effects during testing.

### 2.9. Congo Red Conformation Test

A polysaccharide solution of 1 mg/mL was prepared and mixed with 0.25 mM of Congo red reagent at a volume ratio of 1:1. Appropriate volumes of NaOH solution were added to achieve final concentrations of 0, 0.1, 0.2, 0.3, 0.4, and 0.5 mol/L. Full-wavelength scanning of the configured solution was conducted in the range of 200–600 nm using a UV-Vis spectrophotometer (Shimadzu Corporation, Kyoto, Japan), and the maximum absorption wavelength was recorded [33].

### 2.10. Detection of Antioxidant Activity

#### 2.10.1. Hydroxyl Radical (·OH) Scavenging Activity Detection

PAOR-1 and PAOR-2 solutions with different concentration gradients (0.1, 0.2, 0.4, 0.8, 1.6, and 3.2 mg/mL) were selected, and 1 mL of solution was added to different reaction vessels, followed by 1 mL of ferrous sulfate solution, 1 mL of 5 mmol/L hydrogen peroxide solution, and 1 mL of salicylic acid–ethanol solution. A blank tube was treated with distilled water (1 mL) as the control, and the reaction was carried out in an oven at 37 °C for about 25 min. The absorbance at 510 nm was determined using a UV-Vis spectrophotometer after the reaction mixture was cooled to room temperature. With the same concentration gradient of vitamin C as a positive control, each experiment was repeated three times. The scavenging rate of two polysaccharides on ·OH was calculated according to Formula (1) [34].

(1) $$E\;(\%)=\frac{A_{0}-A_{1}}{A_{0}}\;\times \;100\%$$

*A*_0_: The absorbance of the blank control;

*A*_1_: The absorbance of different concentrations of polysaccharides.

#### 2.10.2. DPPH· Radical Scavenging Activity Detection

PAOR-1 and PAOR-2 solutions with different concentration gradients (0.1, 0.2, 0.4, 0.8, 1.6, and 3.2 mg/mL) were selected. A total of 1 mL of different-concentration solutions was placed in the EP tube and 1 mL of 0.3 mmol/L DPPH solution was added. The blank tube was treated with distilled water (1 mL) as a control. After fully mixing and avoiding light for 1 h, the UV absorbance value was measured at 517 nm [33]. With the same concentration gradient of Vc as the positive control, each experiment was repeated three times. The DPPH· free radical scavenging rate was calculated using Equation (1), where *A*_0_ refers to the absorbance of the blank control and *A*_1_ refers to the absorbance of different concentrations of polysaccharides. 

#### 2.10.3. ABTS· Radical Scavenging Activity

PAOR-1 and PAOR-2 solutions with different concentration gradients (0.1, 0.2, 0.4, 0.8, 1.6, and 3.2 mg/mL) were selected. A total of 1 mL of the sample solution was placed in an EP tube, and 3 mL of the ABTS–potassium persulfate mixture was added. The blank tube used distilled water (1 mL) as a control. Absorbance at 734 nm was measured after the reaction at 37 °C for 1 h in the dark [35]. With the same concentration gradient of Vc as the positive control, each experiment was repeated three times. The ABTS· free radical scavenging rate was calculated using Equation (1), where *A*_0_ refers to the absorbance of the blank control and *A*_1_ refers to the absorbance of different concentrations of polysaccharides.

#### 2.10.4. O_2_^−·^ Radical Scavenging Activity

PAOR-1 and PAOR-2 solutions with different concentration gradients (0.1, 0.2, 0.4, 0.8, 1.6, and 3.2 mg/mL) were selected. A total of 0.2 mL of samples was taken and placed in an EP tube, and 3 mL of Tris-HCl buffer was added. The blank tube used distilled water (0.2 mL) as the control. After mixing, the reaction was carried out at 37 °C for about 15 min. Then, 15 μL of a 30 mmol/L pyrogallol solution was added, and the reaction was carried out for 4 min. Finally, 0.5 mL of concentrated HCl was added quickly to terminate the reaction. Finally, the absorbance of the reaction solution at 325 nm was determined [36]. With the same concentration gradient of Vc as the positive control, each experiment was repeated three times. The scavenging rate of the two polysaccharides on O_2_^−·^ was calculated using Equation (1), where *A*_0_ refers to the absorbance of the blank control and *A*_1_ refers to the absorbance of different concentrations of polysaccharides.

#### 2.10.5. Reducing Capacity Detection

PAOR-1 and PAOR-2 solutions with different concentration gradients (0.1, 0.2, 0.4, 0.8, 1.6, and 3.2 mg/mL) were selected, and 1 mL of samples was taken and placed in an EP tube. Then, 2 mL of 0.2 mol/L phosphate buffer (PBS) and 2 mL of 1% potassium ferricyanide reagent were added and mixed evenly. The blank tube used distilled water (1 mL) as the control. The reaction was then carried out in an oven at 50 °C for about 15 min. After the reaction was completed, it was cooled to room temperature, and then 2.5 mL of a 10% trichloroacetic acid solution and 0.6 mL of a 1% ferric chloride solution were added for oscillation and mixing for 10 min. The reaction was centrifuged at 3000 rpm for 5 min with the same concentration gradient of Vc as the positive control. Each experiment was repeated three times [37]. The reducing capacity was calculated using Equation (1), where *A*_0_ refers to the absorbance of the blank control and *A*_1_ refers to the absorbance of different concentrations of polysaccharides.

#### 2.10.6. Anti-Lipid Peroxidation Capacity Detection

PAOR-1 and PAOR-2 solutions with different concentration gradients (0.1, 0.2, 0.4, 0.8, 1.6, and 3.2 mg/mL) were selected, and 3 mL of samples was taken and placed in EP tubes, followed by 3.5 mL of soybean lecithin (1 mg/mL) and 0.3 mL of FeSO4 (10 mmol/L). The blank tube was treated with distilled water (4 mL) as a control. The mixture was mixed well and then reacted at 37 °C for about 15 min. The reaction was completed by adding 1 mL of trichloroacetic acid solution (20%) and 1 mL of thiobarbituric acid solution (0.8%), and a boiling-water-bath reaction was performed for about 10 min. The absorbance at 535 nm was measured after centrifugation at 3500 rpm for about 5 min [38]. The anti-lipid peroxidation capacity was calculated using Equation (1), where *A*_0_ refers to the absorbance of the blank control and *A*_1_ refers to the absorbance of different concentrations of polysaccharides.

### 2.11. Data Processing and Statistical Analysis

Using SPSS software (Duncan’s multiple range test) to analyze the data, all the data were checked for normality and homogeneity of variance. The Shapiro–Wilk test confirmed a normal distribution (*p* > 0.05) for all groups, justifying the use of one-way ANOVA followed by Duncan’s post hoc test, utilizing SPSS software (version 17.0). A *p*-value of <0.05 was considered statistically significant in comparisons with the blank or model groups.

## 3. Results and Discussion

### 3.1. PAOR Single-Factor Experiment

**Figure 2:**
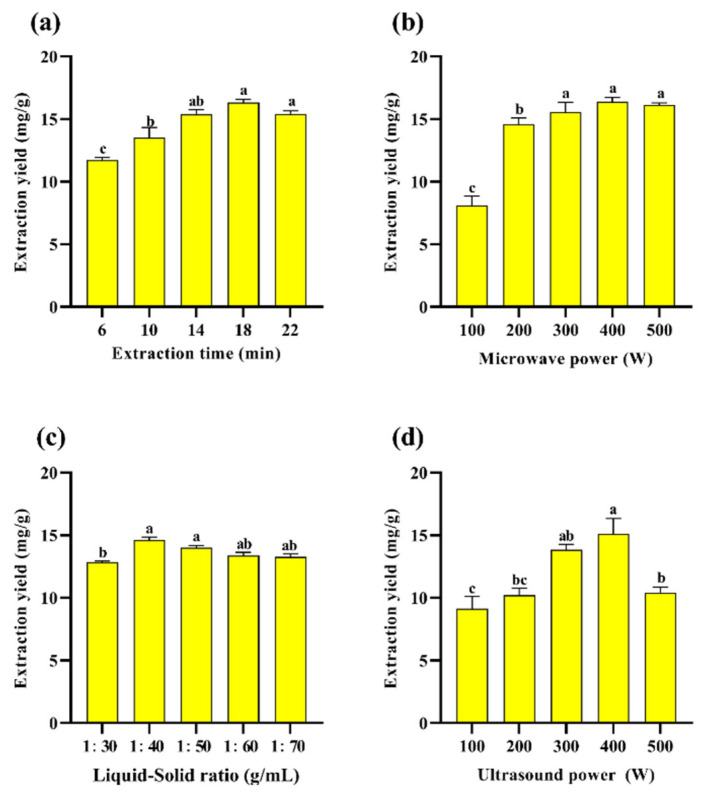
Effect of different (**a**) extraction times; (**b**) microwave power levels; (**c**) liquid–solid ratios; (**d**) ultrasonic power levels on the yield of PAOR. Different lowercase letters indicate significant differences between groups (*p* < 0.05).

Figure 2a–d show the preliminary one-way study of PAOR, where the extraction rate of PAOR was optimized and determined by varying the liquid–solid ratio, extraction time, ultrasonic power, and microwave power. According to Figure 2a, the extraction yield increases from 15.39 ± 0.28% to 16.33 ± 0.31% at extraction times increasing from 14 min to 22 min. After reaching the maximum value at 18 min, followed by a significant decrease after 22 min, which may be attributed to the increase in time, ultrasound favored the diffusion of polysaccharides from the cells into the solution [39], but with the continuous increase in the extraction time, the polysaccharide molecules degraded, and the yield decreased [40,41]. Figure 2b shows the yield of polysaccharides with different microwave power levels. When the microwave power was increased to 500 W, there was still no significant decreasing trend; so, this factor was not selected as the subsequent response surface optimization condition. Figure 2c shows that, in the ultrasonic power range from 300 W to 500 W, the yield of polysaccharides increased from 13.85 ± 0.30% to 15.1 ± 0.23%, and then decreased significantly to 10.42 ± 0.11%, which is probably due to the fact that when the ultrasonic power increased, the mechanical effect and cavitation effect increased to damage the tissues of AOR, which in turn affected the release of polysaccharides from the cells as well as its dissolution [42]. However, when the ultrasonic power is sustained, it may further affect the monosaccharide composition and relative molecular mass of polysaccharides by affecting their structural characteristics, such as the glycosidic bond connection between monosaccharides [43]. Therefore, 300 W to 500 W can be selected as the range for subsequent response surface optimization. Figure 2d shows that the maximum polysaccharide yield of 14.61 ± 0.09% is achieved at a liquid–solid ratio of 1:40 (*v*/*w*); this might be due to the insufficient mixing of the sample with water and the unfavorable release of intracellular polysaccharides into the water [44]. However, as the liquid–solid ration increases, the overall ultrasonic energy per unit volume decreases during UMAE extraction, which reduces the penetration ability of an ultrasound in the solute and solvent [40], resulting in a decrease in the yield of polysaccharides [45].

### 3.2. RSM Results and ANOVA

Based on the outcome of the one-way test, we incorporated three critical extraction parameters as independent variables and the polysaccharide extraction yield as the response variable. To confirm the accuracy of the model, the established mathematical model and its regression coefficients were subjected to ANOVA, and the results are shown in Table 3. From the table, it is easy to see that the regression model reached a highly significant level (*p* < 0.0001), with a good fit (R^2^ = 0.9848). The fitted equation also better reflects some relationships between the three factors on the extraction rate (Adj R^2^ = 0.9651). In addition, the initial primary terms, namely A, B, and C, along with the secondary terms BC, A^2^, B^2^, and C^2^, demonstrated extremely significant effects (*p* < 0.01). The term AC showed a significant impact (*p* < 0.05), whereas only the difference associated with AB was not significant (*p* > 0.05). Based on the F-value, it can be ascertained that the order of factors influencing this polysaccharide is C (ultrasonic power) > A (material–liquid ratio) > B (extraction time). The lack-of-fit value (Table 3) was non-significant (*p* > 0.05), suggesting that the model errors were primarily due to random variations rather than systematic discrepancies. This non-significant lack of fit confirms that the developed model is reliable for predicting the polysaccharide yield under the tested extraction conditions. In conclusion, the established model is suitable for the analysis and prediction of the yield of polysaccharides extracted from *A. officinarum* using the ultrasonic-microwave-assisted technique. Finally, we performed calculations using the following quadratic multinomial regression equation: Yield(%) = 18.35 + 0.46A + 0.27B + 0.55C + 0.061AB − 0.26AC − 0.68BC − 0.31A^2^ − 0.76B^2^ − 0.94C^2^.

**Figure 3:**
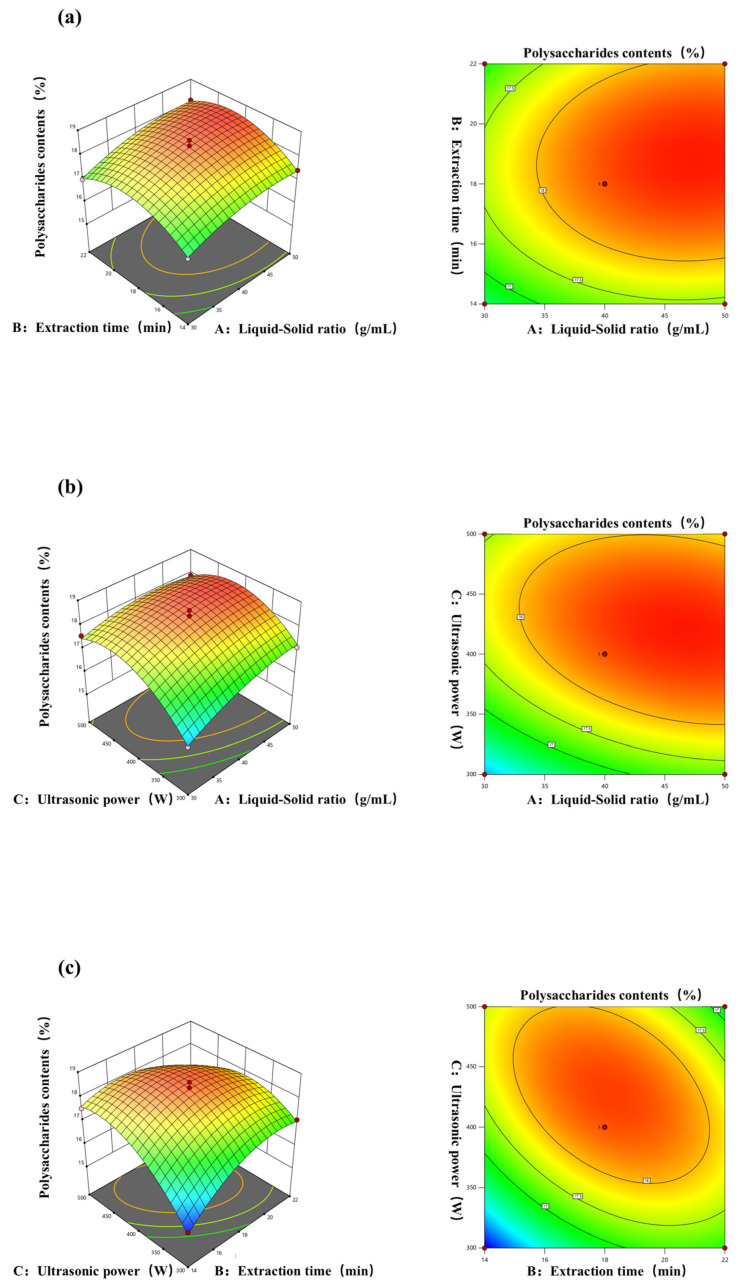
Response surface and contour plots of the effects of (**a**) the liquid–solid ratio and extraction time; (**b**) liquid–solid ratio and ultrasonic power; (**c**) extraction time and ultrasonic power on the yield of polysaccharides from *A. officinarum*.

To optimize variable values in the extraction rate statistical analysis, 3D response surface and 2D contour plots are useful. They vividly show variable behavior at different experimental levels and interactions between pairs of variables. This method aids in pinpointing optimal conditions for each factor when the response value peaks [46]. The impact of the interaction between different yields of polysaccharides by different extraction methods is represented in Figure 3a–c. The experimental results are consistent with the conclusions drawn from the methodological analysis. In Figure 3a, the curves of the liquid–solid ratio and extraction time are similar in steepness, indicating that, when the two interact, there is not much of a difference in the degree of change in the response surface affected by the two factors. As in Figure 3b, when ultrasonic power and the material–liquid ratio interact, the former affects the degree of change in the response surface to a higher degree, and the curve of the ultrasonic power is steeper than that of the extraction time, indicating that when these two factors interact, the ultrasonic power affects the level of change in the response surface to a more sensitive degree [47]. Figure 3c also shows that the polysaccharide yield increases as the ultrasonic power increases, which may be because ultrasonic power enhances solvent penetration into the matrix, thus boosting the polysaccharide yield [48]. This result indicates that when these two factors interact, the ultrasonic power affects the level of change in the response surface to a more sensitive degree.

Based on the above model prediction, the optimal extraction conditions for PAOR-1 were found to be a material–liquid ratio of 1:50 (*v*/*w*), extraction time of 18.704 min, and ultrasonic power of 409.225 W, and the predicted theoretical extraction rate of *A. officinarum* polysaccharide was 18.54 ± 0.45%. To verify the reliability of the predicted response value, the optimized conditions were chosen as a material–liquid ratio of 1:50 (*v*/*w*), extraction time of 19 min, and ultrasonic power of 410 W for three repetitions of the experiment, and the polysaccharide yield obtained was 18.28% ± 2.23%, which was nearly identical to the predicted value, and proved that the optimization result was more reliable.

### 3.3. Purification of Polysaccharides

**Figure 4:**
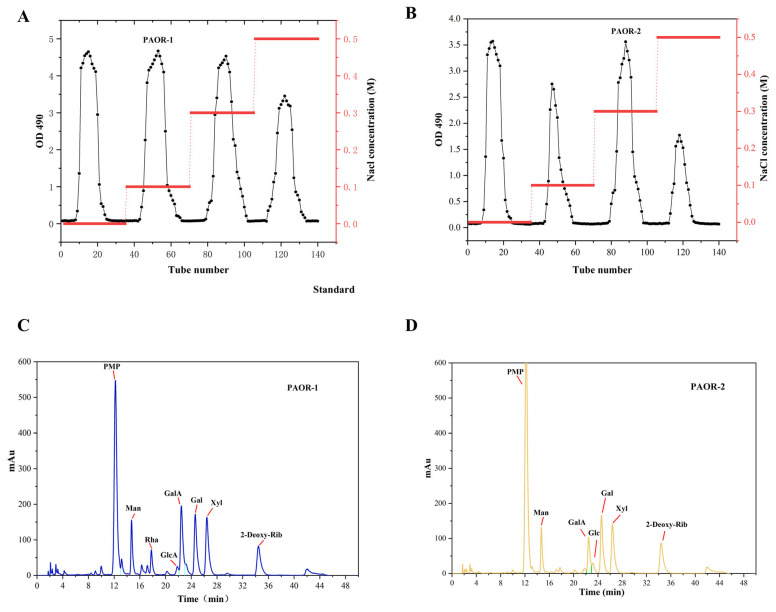
Elution profiles of PAOR-1 (**A**) and PAOR-2 (**B**) purified from the DEAE-52 anion exchange resin column. Monosaccharide composition of PAOR-1 (**C**), and PAOR-2 (**D**).

Dried *A. officinarum* rhizomes were used as the raw material, and hot reflux extraction and ultrasonic-microwave-assisted extraction were employed. Subsequently, through a series of processes, including alcohol precipitation, protein removal by the Sevage method, dialysis, and purification using a DEAE-52 anion exchange resin column [49], two purified polysaccharides were obtained. The elution curves are shown in Figure 4A,B. After vacuum freeze-drying and detection, it was found that the polysaccharides eluted by the 0.1 M NaCl and 0.5 M NaCl solutions had relatively high contents. Therefore, the polysaccharides eluted by the 0.1 M NaCl and 0.5 M NaCl buffer solutions were selected for UMAE and HRE, respectively. These polysaccharides were named PAOR-1 and PAOR-2, respectively, and the subsequent discussion will focus on these two polysaccharides. 

### 3.4. Structural Analysis of Polysaccharide Fractions

#### 3.4.1. Chemical Composition

Comparing the PAOR-1 and PAOR-2 chemical compositions, it was found that (Table 4) the extraction rate of UMAE was significantly higher than HRE. Specifically, the carbohydrate content in PAOR-1 was greater than that in PAOR-2. Statistical analysis revealed that the data between the two groups were significantly different (*p* < 0.05), indicating the superiority of UMAE for polysaccharide extraction with respect to both the extraction rate and carbohydrate content.

#### 3.4.2. Monosaccharide Composition

As depicted in Figure 4C,D and Table 4, PAOR-1 contains D-Mannose, L—Rhamnose, D—Glucuronic acid, D—Galacturonic acid, D—Glucose, D—Galactose, and D—Xylose, with molar ratios of 14.69%, 8.72%, 4.42%, 22.88%, 4.15%, 25.55%, and 19.58%. PAOR-2 contained D-Mannose, D—Galacturonic acid, D—Glucose, D—Galactose, and D—Xylose, with molar ratios of 17.75%, 16.37%, 6.65%, 35.19%, and 24.13%. From these results, we can see that PAOR-1 contains two additional monosaccharides (L-rhamnose and D-glucuronic acid) compared to PAOR-2, with similar compositions of remaining monosaccharides. The results of monosaccharide composition show that both PAOR-1 and PAOR-2 contain more uronic acids. It is worth noting that there are more glucuronic acids in PAOR-1 than in PAOR-2, suggesting that the monosaccharide composition of polysaccharides obtained by the UMAE method is higher, which suggests that, during the hot-water-extraction process, the long-term high temperature may leads to the degradation of certain monosaccharides. The content of D-Galactose in both polysaccharides was relatively high, which proved that the two polysaccharides were acidic galactopolysaccharides [39]. 

### 3.5. FT-IR and UV Analyses

**Figure 5:**
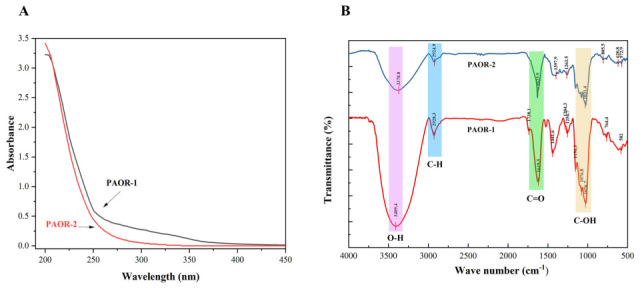
The UV analysis (**A**) and FT-IR analysis (**B**) of PAOR−1 and PAOR−2.

Due to the presence of some aromatic amino acids and the chemical properties of nucleic acids, it is generally believed that proteins and nucleic acids have a certain UV absorption peak in the range of 260–280 nm [50]. As is shown in Figure 5A, through the UV detection of PAOR-1 and PAOR-2, it was found that the two polysaccharides had no obvious absorption peak at 260–280 nm, which indicates that the two polysaccharides contain low levels of protein. However, the absorbance values (0.2–0.4) in the 260–280 nm range suggest that trace amounts of protein may still be present. In Figure 5B, the FT-IR spectra of PAOR-1 and PAOR-2 display similar profiles, both exhibiting characteristic polysaccharide signals. The broad absorption band at 3600–3300 cm^−1^ arises from O–H stretching vibrations, whereas the peaks in the 2900–2800 cm^−1^ region correspond to C–H stretching modes. An absorption band at 1738 cm^−1^, assigned to the C=O stretching vibration, was detected in PAOR-1 but not in PAOR-2, indicating the possible presence of glucuronic acid in PAOR-1, which is consistent with the monosaccharide composition results [51]. Both PAOR-1 and PAOR-2 showed absorption bands at around 1620 cm^−1^, indicating the presence of C=O groups. This confirmed their acidic nature and suggested a high content of galacturonic acid in both polysaccharides. This observation is consistent with our monosaccharide composition analysis. Additionally, the signals between 1200 cm^−1^ and 1000 cm^−1^ are associated with the stretching vibrations of C-O-C and C-O-H bonds [52,53]. The weak peak near 1100 cm^−1^ indicates PAOR-1 and PAOR-2 both have a pyranose ring structure [54]. The peak around 760–800 cm^−1^ reflects the characteristic deformation vibration of pyranose glycosidic bonds [55]. FT-IR analysis revealed that the two polysaccharides predominantly contain pyranose rings and exhibit characteristic features of acidic polysaccharides with β-glycosidic linkages.

### 3.6. Thermal Stability Properties

**Figure 6:**
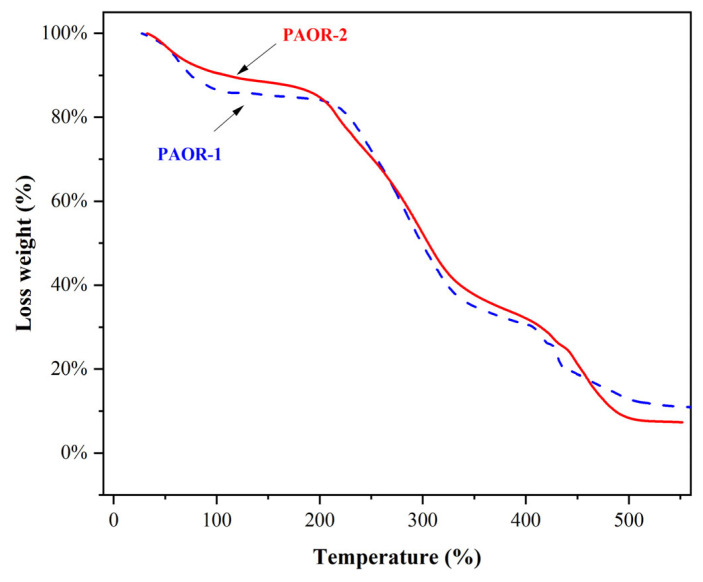
The thermal stability properties of PAOR-1 and PAOR-2.

The thermogravimetric curves of PAOR-1 and PAOR-2 were determined by TGA, as shown in Figure 6. It was found that the heat loss of the two polysaccharides was mainly concentrated in three stages. The first stage was in the range of 30–200 °C, which may be due to the water loss in the two polysaccharides. The inflection point of water evaporation of PAOR-1 was earlier than that of PAOR-2. In the second stage, in the range of 200–400 °C, both PAOR-1 and PAOR-2 had a sharp weight loss, which may be because the structure of the polysaccharides began to depolymerize at this temperature [56]. In the third stage, the degree of thermal decomposition of PAOR-1 and PAOR-2 decreased, and the heat loss rate of PAOR-1 was slightly higher than that of PAOR-2 at the end, which indicated that the thermal stability of polysaccharides extracted by the UMAE method was slightly better than that of HRE [57].

### 3.7. Congo Red Test

**Figure 7:**
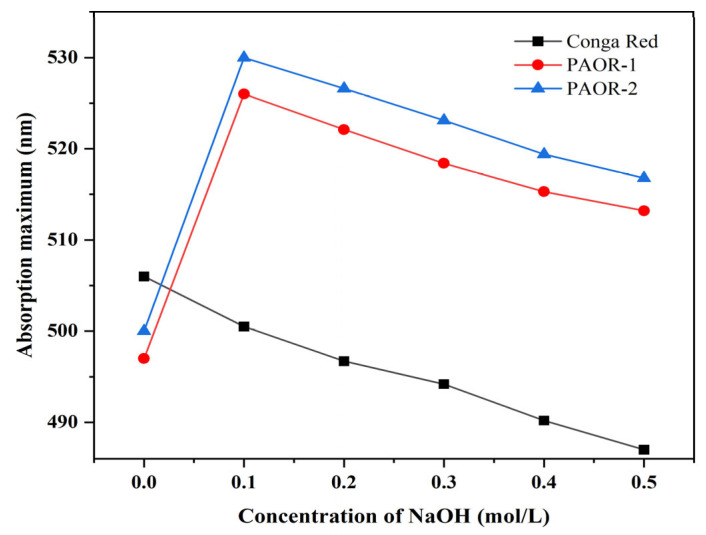
Congo red tests of PAOR-1 and PAOR-2.

Polysaccharides can induce a change in the maximum visible-light absorption of Congo red. Therefore, this method is often used to determine whether polysaccharides have advanced structures by combining the maximum visible-light absorption wavelength of the mixed system in the presence of different concentrations of NaOH [58]. As is depicted in Figure 7, as the NaOH concentration increases to 0.1 M, the λmax values of both PAOR-1 and PAOR-2 show an initial increase. However, at NaOH concentrations exceeding 0.2 M, a gradual decrease in λmax is observed. In contrast, the Congo red control group exhibited a consistent decrease in λmax with an increasing NaOH concentration. These results suggest that PAOR-1 and PAOR-2 possess triple-helical tertiary structures [59].

### 3.8. Molecular-Weight Analysis

**Figure 8:**
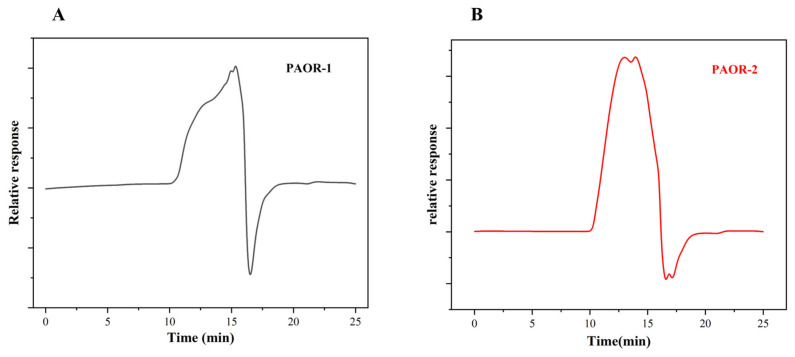
Chromatograms of PAOR-1 (**A**) and PAOR-2 (**B**) by GPC-HPLC.

Studies have shown that the relative molecular mass of polysaccharides has a certain correlation with their biological activity [60]. A standard curve was constructed using dextran standards (180–300,600 Da) with log (Mw) as the x-axis and the retention time as the y-axis, yielding the linear equation y = −2.2847x + 22.874 (R^2^ = 0.9916). The molecular weights of PAOR-1 and PAOR-2 were calculated from this curve, revealing significant disparities in their molecular-weight distributions. As shown in Figure 8A,B and Table 1, only one peak is detected in PAOR-1, with a retention time of 15.08 min, which proves that the polysaccharide has good uniformity. For PAOR-2, two peaks emerged in the elution curve, with retention times of 13.003 min and 13.948 min. Based on the retention times and the standard curve, the relative molecular masses corresponding to the peaks were calculated. The relative molecular mass of PAOR-1 is about 2.6 kDa, while the two peaks of PAOR-2 are calculated to be about 29 kDa. This phenomenon is probably caused during the process of HRE; polysaccharides may accumulate to form certain macromolecules due to high temperatures and long exposure times, and the relative molecular mass also increases [61]. Studies have shown that the antioxidant activity of polysaccharides with a lower molecular weight is better than that of polysaccharides with a higher molecular weight [62].

### 3.9. Surface Microstructural Analysis

**Figure 9:**
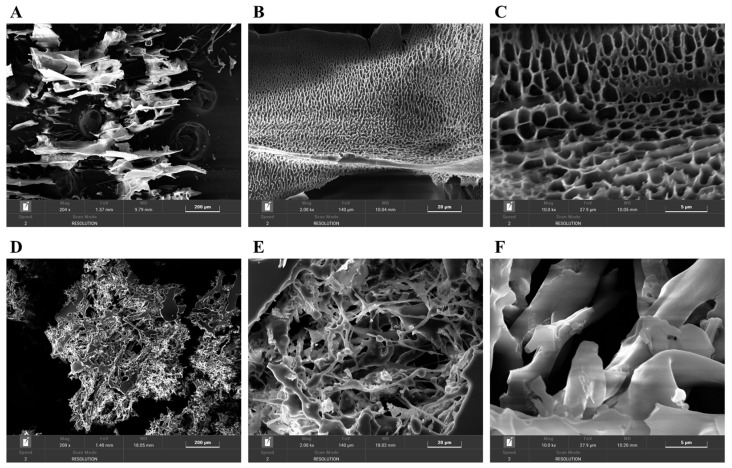
The SEM morphological photos of PAOR-1 (**A**–**C**) and PAOR-2 (**D**–**F**) at 200×, 2000×, and 10,000× magnifications.

SEM was used to observe the microstructures of PAOR-1 and PAOR-2 at different magnifications. In Figure 9A–F, some differences exist in the surface structure of PAOR-1 and PAOR-2. PAOR-1 shows a regular and uniform pore structure at different magnifications (Figure 9A–C), which may be due to the cavitation during the ultrasound process [63]. The structure distribution of PAOR-2 is more scattered (Figure 9D–F), and the polysaccharides can also be observed to have a certain chain structure at a magnification of 2000×, and its combination is more tight. Different structures indicate that the surface structure characteristics of polysaccharides obtained by different extraction methods are quite different. 

### 3.10. AFM Analyses of PAOR-1 and PAOR-2

**Figure 10:**
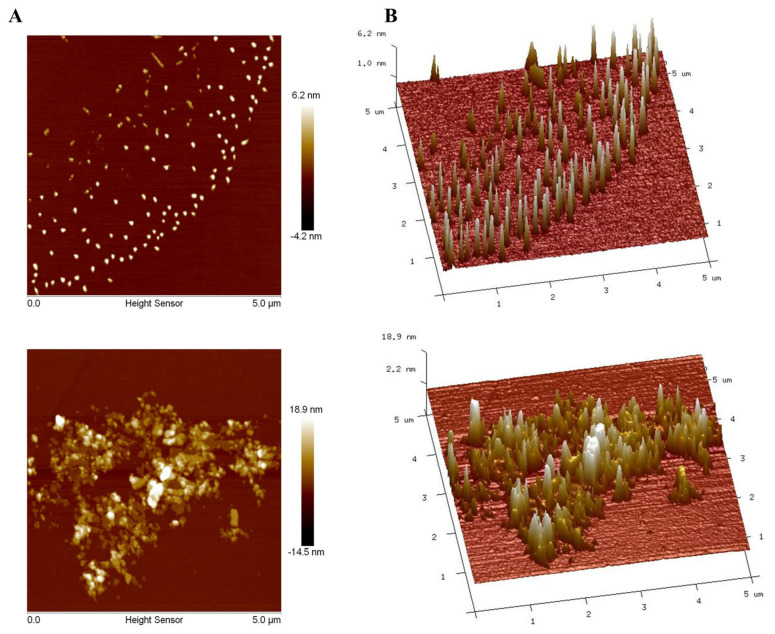
AFM morphological photos of PAOR−1 (**A**) and PAOR−2 (**B**).

To further explore the morphological characterstics of PAOR-1 and PAOR-2, atomic force microscopy (AFM) was used to observe their microstructure. Figure 10A,B reveal that both PAOR-1 and PAOR-2 adopt irregular sheet-like monomeric structures, respectively, with heights varying between 0 and 18.9 nm, alongside elliptically shaped morphologies exhibiting non-uniform dispersion. Scanning electron microscopy (SEM) analysis demonstrated that PAOR-1 possesses a well-ordered porous architecture, whereas PAOR-2 exhibits a more heterogeneous and dispersed structural arrangement. Atomic force microscopy (AFM) further confirmed these observations, revealing a uniformly organized surface topography for PAOR-1 at the nanoscale, in contrast to the loosely aggregated morphology of PAOR-2. Collectively, these multi-scale structural analyses (SEM for microstructural evaluation and AFM for nanoscale resolution) substantiate the distinct structural characteristics of the two polysaccharides, which can be attributed to their differing extraction protocols.

### 3.11. Antioxidant Activity In Vitro

**Figure 11:**
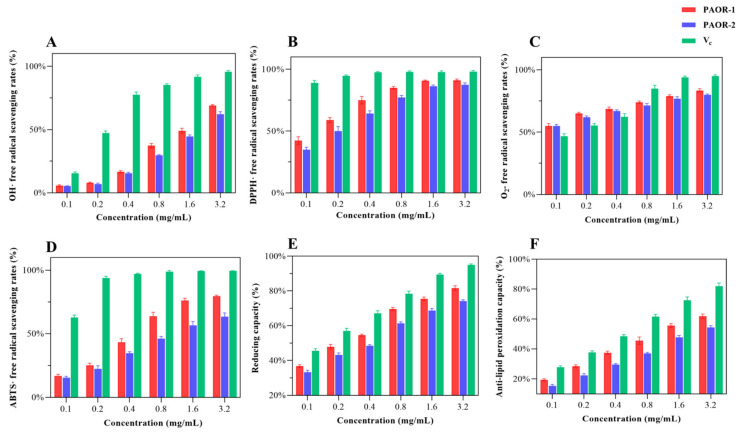
The antioxidant activity PAOR-1 and PAOR-2. (**A**) OH· radical scavenging ability; (**B**) DPPH radical scavenging ability; (**C**) O_2_^−.^ radical scavenging ability; (**D**) ABTS· radical scavenging ability; (**E**) reducing capacity; (**F**) anti-lipid peroxidation ability.

Recent research has confirmed that polysaccharides exhibit various biological activities [7], like inhibiting tumor growth, resisting viral infection, regulating blood glucose levels, scavenging free radicals, and regulating immune responses. Among them, the antioxidant properties of polysaccharides are particularly noticeable. When the intracellular ROS concentration exceeds the physiological threshold, it leads to the destruction of redox balance [64]; free radical chain reaction activation; and irreversible damage of biological macromolecules [65]. This pathological mechanism has prompted researchers to explore the antioxidant application value of polysaccharides. As a natural active ingredient, polysaccharides are widely used in the fields of medicine and functional foods due to their significant free radical scavenging ability [66]. In this study, a multi-dimensional evaluation system (·OH, DPPH·, ABTS·, and O_2_^−·^ scavenging rate; reducing capacity; anti-lipid peroxidation capacity) was innovatively established to provide a standardized evaluation scheme for the antioxidant activity of polysaccharides from different extraction processes [67]. In Figure 11A, the hydroxyl radical scavenging ability of PAOR-1 and PAOR-2 is weak at low concentrations, which is lower than the OH· scavenging ability of vitamin C (Vc). With the continuous increase in concentration, the OH · scavenging ability of the two polysaccharides also shows a certain improvement. From Figure 11B, it can be seen that the two polysaccharides show a good DPPH radical scavenging ability at low concentrations. When the drug concentration keeps rising, there is no notable disparity in the antioxidant ability between PAOR-1 and Vc (*p* > 0.05). In Figure 11C, the scavenging ability of the superoxide anions of the two polysaccharides is higher than that of Vc at a low concentration, but with the increase in the concentration to 0.8 mg/mL, the scavenging ability of O_2_^−.^ is lower than that of Vc. After the concentration reached 1.6 mg/mL, the scavenging ability of the two polysaccharides to O_2_^−.^ tended to be stable. In Figure 11D, the scavenging ability of PAOR-1 and PAOR-2 to ABTS· was enhanced in a concentration-dependent manner. The scavenging rate of PAOR-1 at the highest concentration was significantly higher than that of PAOR-2, but the antioxidant activity of both was lower than that of vitamin C at all concentrations. In Figure 11E, both PAOR-1 and PAOR-2 have a drug-dependent-reducing capacity, but this ability is weaker than Vc. As shown in Figure 11F, PAOR-1 and PAOR-2 show weak anti-lipid peroxidation activity in the range of 0–1.6 mg/ mL, and their clearance rate is significantly lower than that of the vitamin C control group (*p* < 0.05). Even at the highest test concentration (3.2 mg/mL), the activity of the two reached only the half-effective concentration (EC_50_) threshold level.

In general, the antioxidant capacity of the polysaccharides obtained by the UMAE method is stronger than that of PAOR-2, which may be related to the fact that PAOR-1 contains more glucuronic acid and galacturonic acid [68]. Secondly, for the relative molecular mass of the two, the lower molecular weight of PAOR-1 is consistent with the rule that the low-molecular-weight polysaccharides reported in previous studies have better antioxidant activity [69]. Secondly, it is reasonable to speculate that the extraction process of the UMAE method is relatively mild, avoiding high temperatures and a long extraction time, which may make the structure of polysaccharides more complete and regular, which can also be seen in the scanning electron microscope images. Therefore, the antioxidant capacity of polysaccharides extracted via UMAE was greater than that of the HRE extraction method.

## 4. Conclusions

In this study, the UMAE method was used to optimize the extraction of AOR polysaccharides for the first time. The results of RSM show that the optimal extraction conditions are a liquid–solid ratio of 1:50, extraction time of 19 mins, and ultrasonic power of 410 W. In these conditions, the expected polysaccharide yield was 18.28% ± 2.23%, which was significantly higher than that of the traditional hot-water-extraction method. In the future, we will commit to performing comparative studies with ultrasound-assisted extraction and supercritical fluid extraction. The polysaccharides obtained by the two methods were further purified to obtain PAOR-1 and PAOR-2. Structural characterization showed that the two polysaccharides belonged to acidic galactopolysaccharides and had a triple-helix spatial structure. Further studies have found that different extraction methods lead to differences in yield, structural characteristics, and antioxidant activity. PAOR-1 and PAOR-2 have different monosaccharide compositions, surface morphologies, and thermal stability levels. The biological activity indicates that PAOR-1 may have better antioxidant activity than PAOR-2 due to its high uronic acid level, lower molecular weight, and more closely arranged spatial configuration. Therefore, it can be concluded that UMAE seems to be a more suitable choice for a better yield and better biological activity than HRE. However, the quality feature comparison of other extraction methods and further purification to obtain more detailed structural features still need to be further explored.

## Figures and Tables

**Table 1:** Factor levels.

Factor Levels		Independent Variable	
	Material–Liquid Ratio (*v*/*w*)	B Extraction Time (min)	C Ultrasonic Power (W)
−1	30	40	50
0	14	18	22
1	300	400	500

**Table 2:** The design and results of the ultrasonic-microwave-assisted extraction responses.

No.	Factor			Yield (%)
	A/(mL/g)	B/(min)	C/(W)	
1	30	14	400	16.55
2	50	14	400	17.43
3	30	22	400	16.98
4	50	22	400	18.15
5	30	18	300	15.78
6	50	18	300	17.14
7	30	18	500	17.56
8	50	18	500	17.89
9	40	14	300	15.23
10	40	22	300	17.12
11	40	14	500	17.53
12	40	22	500	16.69
13	40	18	400	18.63
14	40	18	400	18.11
15	40	18	400	18.41
16	40	18	400	18.33
17	40	18	400	18.25

Note: A corresponds to the material–liquid ratio (*v*/*w*), B corresponds to the extraction time (min), and C corresponds to ultrasonic power (W).

**Table 3:** ANOVA results for the regression equation.

Source of Variation	Square Sum	Degrees of Freedom	Mean Square	F-Value	*p*-Value	Significance
Model	14.08	9	1.56	50.23	<0.0001	**
A	1.72	1	1.72	55.07	0.0001	**
B	0.59	1	0.59	18.80	0.0034	**
C	2.42	1	2.42	77.67	<0.0001	**
AB	0.015	1	0.015	0.48	0.5101	ns
AC	0.27	1	0.27	8.51	0.0224	*
BC	1.86	1	1.86	59.80	0.0001	**
A^2^	0.42	1	0.42	13.40	0.0081	**
B^2^	2.46	1	2.46	79.06	<0.0001	**
C^2^	3.71	1	3.71	119.06	<0.0001	**
Residual	0.22	7	0.031			
Lack of fit	0.068	3	0.023	0.61	0.64	ns
Pure error	0.15	4	0.037			
Total error	14.30	16				
	R^2^ = 0.9848		R_adj_^2^ = 0.9651		C.V.% = 1.01	


Note: “*” represents a significant difference, *p* < 0.05; “**” represents a highly significant difference, *p* < 0.01; “ns” represents a difference that is not significant.

**Table 4:** Chemical composition of PAOR-1 and PAOR-2.

Item	PAOR-1	PAOR-2
**Yield (%)**	17.56 ± 1.62% ^a^	10.12 ± 3.45% ^b^
**Chemical characteristics (%)**		
Carbohydrate (%)	84.12 ± 0.25% ^a^	78.4 ± 0.65% ^b^
Protein (%)	1.14 ± 0.32% ^a^	0.65 ± 0.15% ^a^
**Molecular weight (kDa)**		
Peak1 Mw (kDa)	2.6	20.9
Peak2 Mw (kDa)	ns	8.1
**Monosaccharide composition (mol%)**		
D-Mannose	14.69	17.75
D-Glucosamine	ns	ns
L-Rhamnose	8.72	ns
D-Glucuronic acid	4.42	ns
D-Galacturonic acid	22.88	16.37
D-Glucose	4.15	6.65
D-Galactose	25.55	35.19
D-Xylose	19.58	24.13

Note: “ns” indicates that the item was not found. Data are shown as mean ± SD (*n* = 3). Different letters indicate significant differences (*p* < 0.05).

## Data Availability

Data are available upon request.
